# Physicochemical characterization of *Pseudomonas stutzeri* UFV5 and analysis of its transcriptome under heterotrophic nitrification/aerobic denitrification pathway induction condition

**DOI:** 10.1038/s41598-020-59279-7

**Published:** 2020-02-10

**Authors:** Lívia Carneiro Fidélis Silva, Helena Santiago Lima, Tiago Antônio de Oliveira Mendes, Adilson Sartoratto, Maira Paula Sousa, Rodrigo Suhett de Souza, Sérgio Oliveira de Paula, Valéria Maia de Oliveira, Cynthia Canedo Silva

**Affiliations:** 1 Department of Microbiology, Federal University of Viçosa, Viçosa, Minas Gerais 36570-900 Brazil; 2 Departament of Biochemistry, Federal University of Viçosa, Viçosa, Minas Gerais 36570-900 Brazil; 3 Pluridisciplinary Center for Chemical, Biological and Agricultural Research, Campinas State University, Campinas, São Paulo 13083-970 Brazil; 4 Petrobras - Research and Development Center (CENPES), Petrobras, Rio de Janeiro, 21941-915 Brazil; 5 Department of General Biology, Federal University of Viçosa, Viçosa, Minas Gerais 36570-900 Brazil

**Keywords:** Water microbiology, Environmental impact

## Abstract

Biological ammonium removal via heterotrophic nitrification/aerobic denitrification (HN/AD) presents several advantages in relation to conventional removal processes, but little is known about the microorganisms and metabolic pathways involved in this process. In this study, *Pseudomonas stutzeri* UFV5 was isolated from an activated sludge sample from oil wastewater treatment station and its ammonium removal via HN/AD was investigated by physicochemical and molecular approaches to better understand this process and optimize the biological ammonium removal in wastewater treatment plants. Results showed that *P. stutzeri* UFV5 removed all the ammonium in 48–72 hours using pyruvate, acetate, citrate or sodium succinate as carbon sources, C/N ratios 6, 8, 10 and 12, 3–6% salinities, pH 7–9 and temperatures of 20–40 °C. Comparative genomics and PCR revealed that genes encoding the enzymes involved in anaerobic denitrification process are present in *P. stutzeri* genome, but no gene that encodes enzymes involved in autotrophic nitrification was found. Furthermore, transcriptomics showed that none of the known enzymes of autotrophic nitrification and anaerobic denitrification had their expression differentiated and an upregulation of the biosynthesis machinery and protein translation was observed, besides several genes with unknown function, indicating a non-conventional mechanism involved in HN/AD process.

## Introduction

Nitrogen is considered to be one of the main pollutants of industrial effluents, and its presence can cause the eutrophication of the environments where they are discharged^1,2^. Conventionally, biological ammonium removal from wastewater treatment stations occurs through two sequential and independent processes: autotrophic nitrification followed by anaerobic denitrification, where the first stage is carried out by autotrophic bacteria and/or archaeas in aerobiosis, and the second by heterotrophic bacteria in anaerobiosis^3–5^. Due to the differences in the physical and chemical conditions of the two stages, these processes usually occur at separate times in the effluent treatment plants, demanding a longer reaction time and more space. In addition, the autotrophic microorganisms involved in nitrification are of slow growth, making the detention time of the effluent in the reactor even longer.

To overcome these limitations, academic studies have focused on new processes for biological nitrogen removal, and heterotrophic nitrification/aerobic denitrification (HN/AD) has been gaining prominence in the effluent treatment field. In this process, a single microorganism is able to transform ammonium into gaseous nitrogen under aerobic conditions, which makes it advantageous in comparison with the conventional process^6^. Because they are heterotrophic, these microorganisms remove ammonium and organic matter from the effluent, in addition to having faster growth when compared to the autotrophic nitrifiers^7,8^. However, unlike the conventional ammonium removal process, which is already well elucidated, many details of HN/AD are still unclear, and little is known about the metabolism involved in this process as well as the microorganisms capable of performing it. Until now, no specific marker for HN/AD has been found, and studies in literature aiming to investigate this metabolic pathway have relied on the enzymes involved in autotrophic nitrification and anaerobic denitrification and in the intermediates formed during the reactions. However, since most of the HN/AD microorganisms described are also anaerobic denitrifiers, the detection of these enzymes and intermediates alone is not enough to characterize the complete pathway.

Considering the importance of this process for ammonium removal in the treatment of effluents and the lack of information about the microorganisms involved and their metabolism, in this work, an HN/AD isolate was characterized in terms of the influence of physicochemical factors of biological ammonium removal, and its metabolism in an induction condition of the metabolic pathways involved in the HN/AD process was investigate. We believe that a better understanding of the physiology of this microorganism as well as HN/AD-related metabolic pathways may result in an optimization of the operational processes for more efficient biological ammonium removal in effluent treatment stations.

## Results

### Identification and characterization of the isolate UFV5

**Figure 1:**
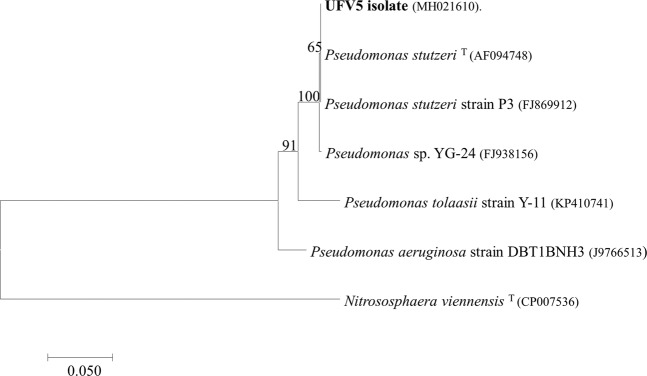
Phylogenetic tree based on the partial sequence of the 16S rRNA of the UFV5 isolate, reference sequence (T) and sequences of HN/AD microorganisms belonging to the same genus described in the literature. The tree was constructed by the neighbor joining method with a bootstrap value represented in the branches referring to 1000 replications. The accession numbers of the GenBank sequences are shown in parentheses. The *Nitrososphaera viennensis* sequence was added as the outgroup.

The alignment of the isolate sequence with the sequences of GenBank database revealed that it is related to members of the genus *Pseudomonas*, with 100% identity with the species *Pseudomonas stutzeri* (accession number KY616652.1). Phylogenetic inference showed that the sequence of the isolate formed a cluster with others sequences of *P. stutzeri* HN/AD described in the literature, including the reference organism sequence (Fig. 1).

**Figure 2:**
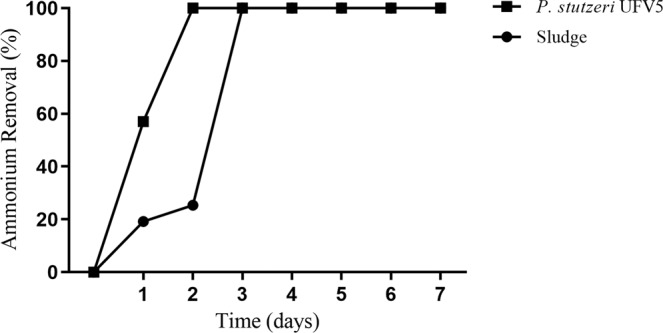
Percentage of ammonium removal from the *P. stutzeri* UFV5 isolate over time. Cultures were incubated in HNM medium for seven days, and ammonium quantification was monitored every 24 hours. Activated sludge was used as a positive control.

*P. stutzeri* UFV5 removed 57.2% ± 2.15 of ammonium within 24 hours of incubation, and in less than 48 hours all ammonium had already been removed, faster than the positive control (activated sludge), which in 24 and 48 hours removed 20,4% ± 1,4 and 25,8% ± 1,8, respectively (mean% ± standard deviation, n = 3) (Fig. 2).

**Figure 3:**
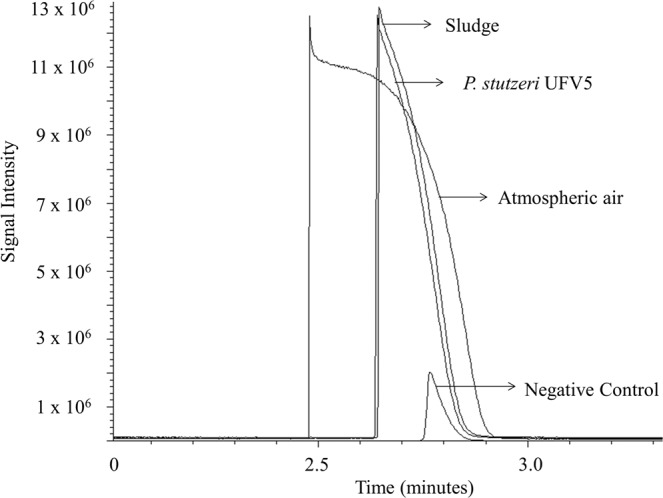
Production of gaseous nitrogen by *P. stutzeri* UFV5 bacteria during the ammonium removal under aerobic conditions. The positive control of the technique was performed by the injection of pure atmospheric air, the positive control of the biological process was HNM medium inoculated with nitrifying activated sludge, and negative control was HNM medium without inoculum. Axis X = time, in minutes, of gas detection, and axis Y = signal intensity, the larger the peak, the greater the amount of gaseous nitrogen produced.

In addition, the isolate was capable of producing gaseous nitrogen as well as activated sludge when incubated in medium for heterotrophic nitrifying under aerobic conditions, validating its ability to remove ammonium by the HN/AD process (Fig. 3). Nitric and nitrous oxide gases were not detected by chromatography.

**Figure 4:**
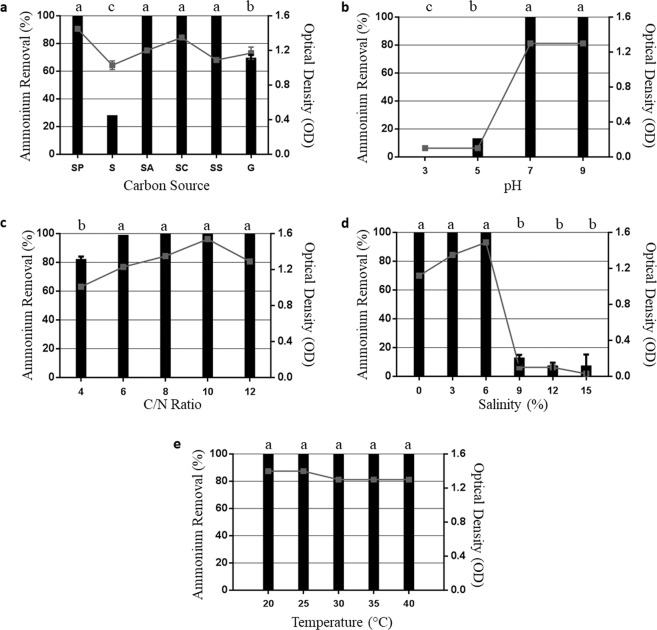
Effect of physical-chemical factors on growth () and ammonium removal (■) of bacterial isolate *P. stutzeri* UFV5 after 72 hours of incubation in HNM medium. (**a**) Carbon source - the carbon sources used were sodium pyruvate (SP), sucrose (S), sodium acetate (SA), sodium citrate (SC), sodium succinate (SS) and glucose (G)), (**b**) pH - 3, 5, 7 and 9, (**c**) Carbon/Nitrogen Ratio - 4, 6, 8, 10 and 12, (**d**) Salinity - 0, 3, 6, 9, 12 and 15% of NaCl and (**e**) Temperature - 20, 25, 30, 35 and 40 °C. The graph represents the mean values of ammonium removal and optical density and their respective standard deviations. Ammonium removal means followed by at least one same letter did not differ at a 5% level of significance as determined by Tukey’s test.

The use of different carbon sources, pH and salinities significantly affected the growth (Fig. 4 and Table S1) and ammonium removal of *P. stutzeri* UFV5. The growth in pyruvate, acetate, citrate and sodium succinate sources reflected in a maximum ammonium removal, while with sucrose and glucose the removal was 30 and 70%, respectively (Fig. 4a). In acidic phs (3 and 5) and very high salinity (9, 12 and 15% NaCl) there was no microbial growth and ammonium removal was very low, reaching 0 at pH 3 (Fig. 4b,d). The temperatures and C/N ratios evaluated did not seem to influence the ammonium removal activity of *P. stutzeri* UFV5 (Fig. 4c,e). There was no change in the pH values of the treatments during the incubation period.

Of all the ammonium removed during the process of HN/AD, approximately 47% was transformed into microbial biomass and 53% was lost as gaseous nitrogen, according to nitrogen balance. The intermediates hydroxylamine, nitrite and nitrate were not detected (Table 1).

**Table 1:** Nitrogen balance of isolate *P. stutzeri* UFV5.

Isolate	Hydroxylamine (mg)	Nitrite (mg)	Nitrate (mg)	Intracellular Nitrogen (% mean ± SD)	Nitrogen Gas (% mean ± SD)
*P. stutzeri* UFV5	ND	ND	ND	47,02 ± 4,90	52,98 ± 4,90

*ND = not detected and SD = standard deviation.

### Investigation of enzymes involved in HN/AD by the molecular approach

The comparative genomics showed that the genes of the anaerobic denitrification process are present in reference genome of the *P. stutzeri* strain CGMCC 1.1803^9^, while those involved in autotrophic nitrification were not detected by sequence alignment (Table 2a). These results were confirmed by PCR for the key genes of conventional ammonium removal in the genome of *P. stutzeri* UFV5. The genes encoding the AmoA and HaoF enzymes involved in autotrophic nitrification were not detected, however, those that encode the enzymes involved in the anaerobic denitrification process were (Table 2b).

**Table 2:** (a) Comparative analysis of the sequences of enzymes involved in with the genome of *P. stutzeri* available in NCBI.

a	Enzymes involved in autotrophic nitrification and anaerobic denitrification process evaluated in comparative genomic analysis									
*P. stutzeri* UFV5	AmoABC	Hao	NarG/NxrA	NarH/NxrB	NarI	NapA	NirS	NirK	NorBC	NosZ
	−	−	+	+	+	+	+	−	+	+
**b**	**Genes that encode enzymes involved in autotrophic nitrification and anaerobic denitrification process analyzed by PCR**									
*P. stutzeri* UFV5	amoA	haoF	nap		nirS		norB		nosZ	
	−	−	+		+		+		+	

The positive (+) sign represents the presence of the gene in the *P. stutzeri* genome and the negative (−) sign represents the absence of the gene. (b) Detection of genes encoding enzymes related to autotrophic nitrification and anaerobic denitrification the process in the genome of the isolate *P. stutzeri* UFV5.

Increasing the ammonium concentration of the medium directly affects the ammonium removal activity: in HNM medium with low ammonium concentration, the consumption within 2 hours after incubation was 1.2 mg, and in high ammonium concentration medium, at the same time interval, 30.0 mg was consumed. Microbial growth is unchanged, under both conditions the optical density increased from 0.1 to approximately 0.2 (Table 3).

**Table 3:** Ammonium consumption and growth of *P. stutzeri* UFV5 in HNM medium with low and high ammonium concentration.

Ammonium concentration in HNM medium (gL^−1^)	Ammonium consumption (mg) (mean ± SD)	Optical density (mean ± SD)
0.16	1.197 ± 0.23	0.244 ± 0.02
1.32	30.04 ± 2.90	0.234 ± 0.04

*SD = Standard deviation.

**Figure 5:**
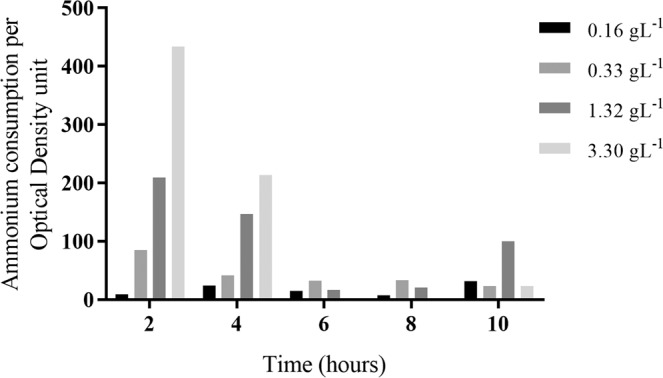
Ammonium consumption by optical density unit (normalized values) of *P. stutzeri* UFV5 at different concentrations of ammonium. Analyses were performed every 2 hours over the 10-hour period.

As ammonium consumption increased with increasing ammonium concentration in the medium and the microbial growth was the same, the ratio of consumption per unit of optical density follows the same profile, showing that the effect is dose-dependent for the first 4 hours after incubation (Fig. 5).

As the concentration of 3.3 gL^−1^ of ammonium is very high and could deviate from the linearity of the standard curve of the colorimetric test, we selected concentrations of 0.16 gL^−1^ and 1.32 gL^−1^ and a time of 2 hours for induction of the HN/AD pathway for comparative transcriptomics analysis.

The ammonium consumption by optical density of the two colonies of *P. stutzeri* UFV5 inoculated for the study of the transcriptome in the lowest ammonium concentration medium was lower than the consumption at the highest concentration medium, indicating that there was induction of the expression of the genes involved in the ammonium removal pathway by HN/AD (Fig. S1). After 2 hours of incubation, all replicates of the two colonies showed more than 99% of living cells, ensuring the quality of RNASeq data: 99.90% ± 0.09 for 0.16 gL^−1^ C1, 99.93% ± 0.03 for 0.16 gL^−1^ C2, 99.87% ± 0.03 for 1.32 gL^−1^ C1 and 99.89% ± 0.04 for 1.32 gL^−1^ C2 (Average ± standard deviation, n = 3).

**Figure 6:**
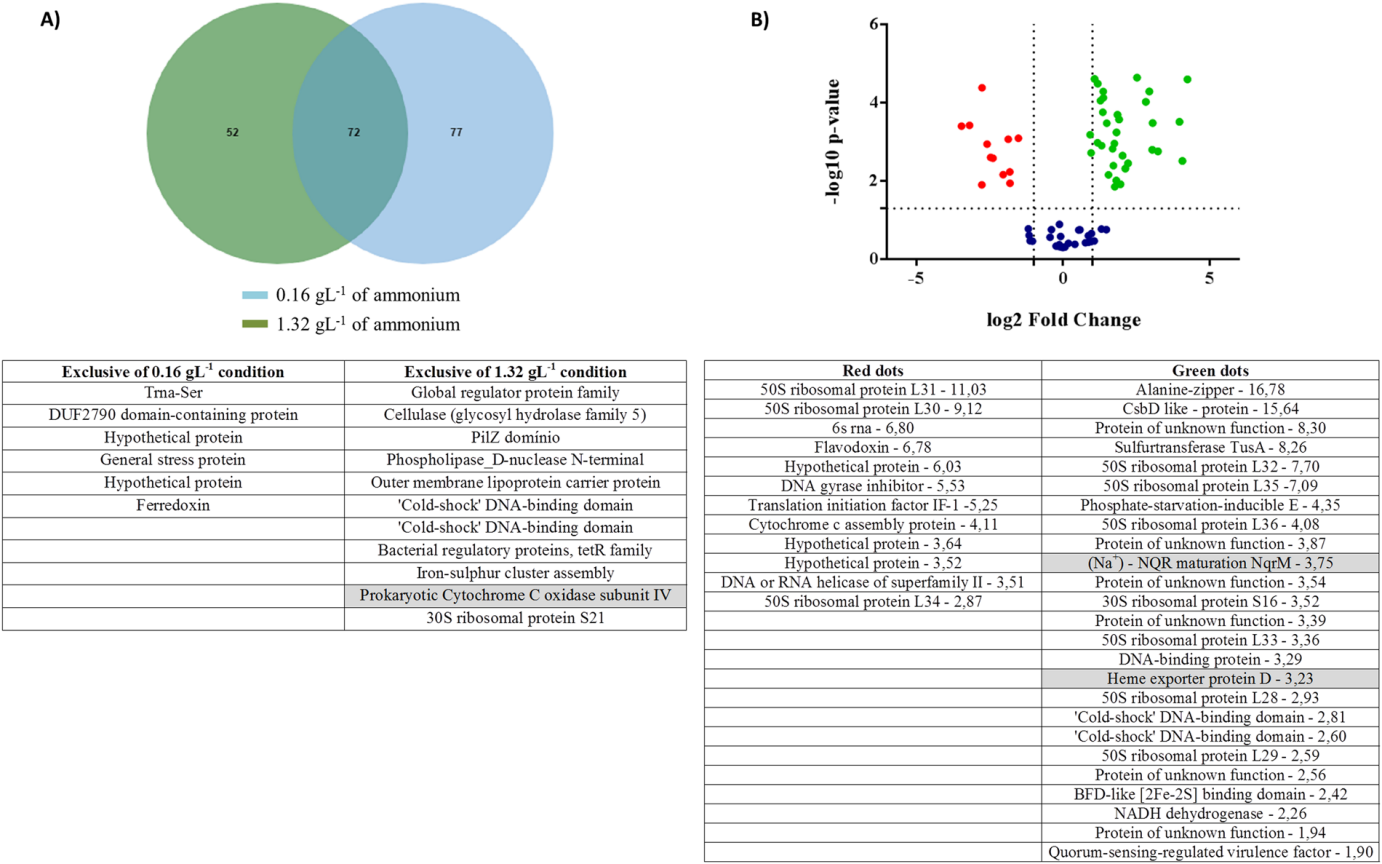
Transcriptome analysis of the isolate *P. stutzeri* UFV5 in low and high concentrations of ammonium. (**A**) Venn diagram showing the number of genes that were expressed specifically in each condition and those expressed in both, and in the table below the figure are described the genes that were expressed with statistical significance exclusively in each condition; (**B**) Volcano plot to show differentially expressed genes regulated negatively and positively in both treatments: red = negatively regulated expression genes with statistical significance; green = positively regulated expression genes with statistical significance; blue = expression genes unaltered, and in the table below the figure are the annotation with fold change of those genes represented by the red and green dots.

Analysis of transcriptome data indicate that biological replicates of *P. stutzeri* UFV5 isolate under low and high ammonium concentrations were similar (Fig. S2). Of the 201 differentially expressed genes, 25.87% were exclusive of the lower ammonium concentration condition, 38.31% exclusive of the higher ammonium concentration condition and 35.82% were shared by both (Fig. 6A, and Tables S2–S4). In the volcano plot analysis (Fig. 6B), it is possible to observe the genes that were differentially expressed with statistical significance between the two treatments (above the dashed line) regulated either positively (green) or negatively (red), considering a value of fold change (the number of times that the gene was more or less expressed in the condition of higher ammonium concentration in relation to that of the lesser concentration) above 2.

Considering the genes with statistical significance, 11 were expressed only in the condition of high ammonium concentration, 6 only in the condition of low ammonium concentration, 29 were expressed in both conditions with positive regulation and 12 with negative regulation (Fig. 6). All genes that are highlighted in the tables participate in oxidation-reduction reactions.

**Figure 7:**
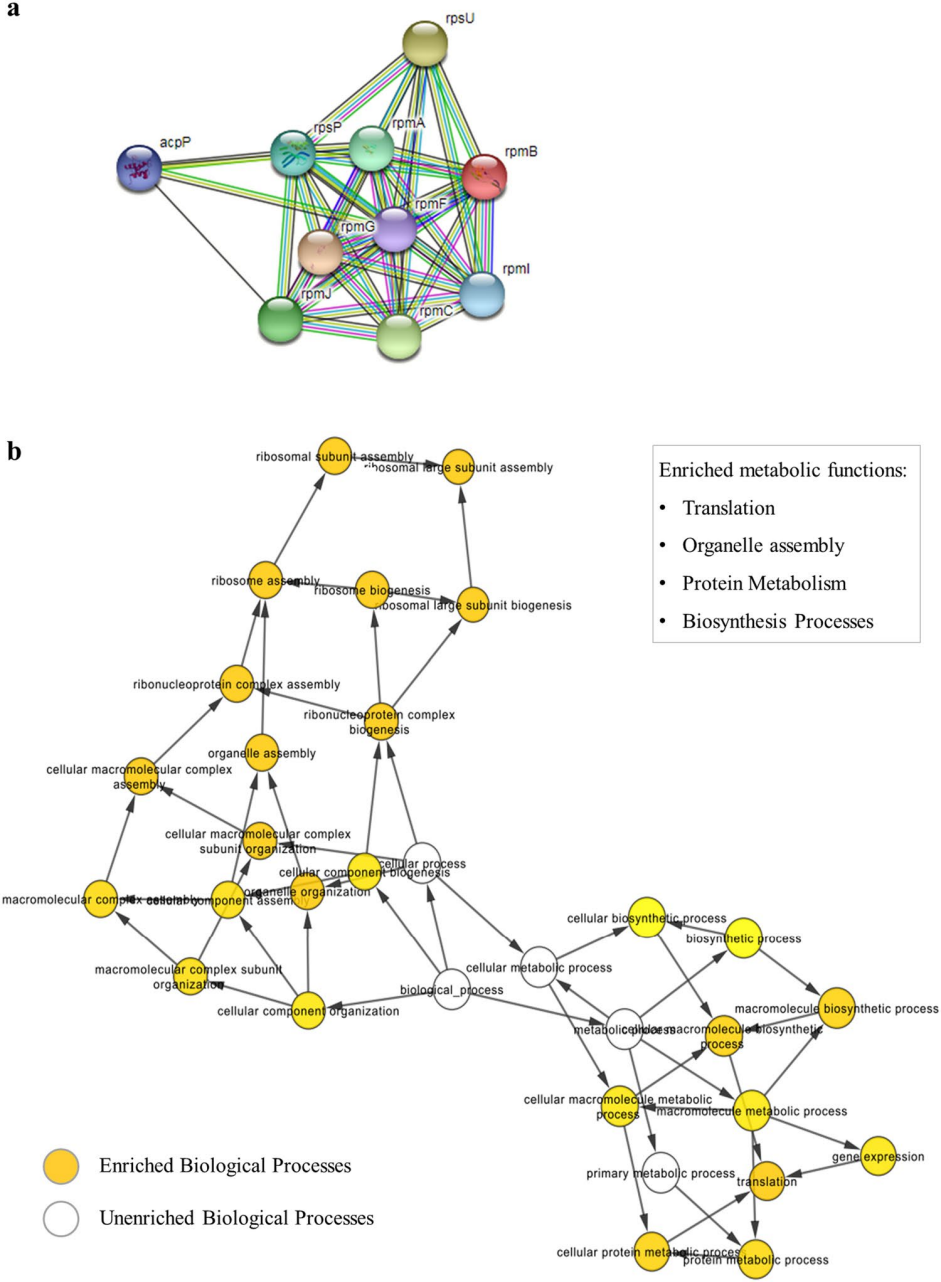
Differentially expressed genes under conditions of low and high ammonium concentrations that were up regulated. (**a**) Network of interactions of the genes based on their functions obtained by the STRING program; (**b**) Enrichment analysis of biological processes with increasing ammonium concentration by the GOanna software. The colored balls indicate the enriched processes - the stronger the yellow, the more enriched the process was, and the white balls indicate unenriched processes that serve as connectors to the network.

The interaction network between genes that were positively regulated showed that those who encoding the Rps and Rpm family proteins were highly enriched during the ammonium induction process (Fig. 7a), and that these proteins are involved with the functions of assembly of cellular organelles, the biosynthesis process, protein metabolism and protein translation (Fig. 7b).

## Discussion

Several species of the genus *Pseudomonas*, such as *P. tolaasii, P. aeruginosa* and also *P. stutzeri*, have been previously described as capable of heterotrophic nitrification/aerobic denitrification, however, through phylogenetic inference, it was observed that the UFV5 isolate differs from those species already described^10–12^.

After identification of the isolate, its ability to perform heterotrophic nitrification/aerobic denitrification was evaluated. *P. stutzeri* UFV5 was able to remove ammonium from a culture medium using an organic carbon source, and transform it into gaseous nitrogen under aerobic conditions, thus demonstrating its HN/AD capability. According to the literature, strains of the genus *Pseudomonas* HN/AD are able to remove ammonium with high efficiency in up to 4 days^10–12^, and the *P. stutzeri* UFV5 isolate was able to remove all ammonium present in the medium within 48 hours, a time period considerably shorter than that of the described isolates. Nitric oxide and nitrous oxide were not detected by chromatography after seven days after inoculation, suggesting two possible reasons: the HN/AD pathway does not pass through these intermediates, or they had already been completely reduced to gaseous nitrogen at the time of analysis.

The response of the *P. stutzeri* UFV5 isolate to the different physicochemical factors was promising, since ammonium removal remained high under most of the evaluated conditions. The lowest values for removal were reached when carbon sources were sucrose and glucose, pH was 3 or 5 and salinity was 9, 12 or 15% of NaCl, and the C/N ratios and temperatures analyzed do not affect ammonium removal efficiency. According to the literature, for the microorganisms that perform the HN/AD process, the preferred sources of carbon are succinate, acetate and citrate, which are directly involved in the tri-carboxylic acid cycle^13^, which explains the lower ammonium removal when carbon sources were glucose and sucrose. Regarding salinity, *P. stutzeri* UFV5 removed 100% of ammonium in a medium with up to 6% NaCl, twice the salinity found in seawater. Effluents arriving at treatment plants may have high salinity, and salinity is known to be a factor that directly affects biological processes including ammonium removal^14,15^, so microorganisms that can perform their functions under such conditions are desirable, and *P. stutezi* UFV5 showed promising results. Results obtained in the literature show that different microorganisms respond in unique ways to physicochemical factors, but the majority of those capable of performing HN/AD have a preference for carbon sources directly involved in the citric acid cycle, a C/N ratio between 8 and 10, pH between 7 and 9, temperature close to 30 °C and do not tolerate increased salinity^8,13,16^. Thus, the results found for *P. stutzeri* UFV5 were promising, since it maintained high ammonium removal efficiency in different conditions of carbon source, C/N ratio, pH, salinity and temperature. Therefore, for application in wastewater treatment plants, this bacterium would be more resilient to shocks that may occur in reactors, possibly conserving its activity to remove ammonium without compromising process efficiency.

Another important factor for wastewater treatment plants is the generation of excess biomass: the excess of sludge generated that is not recirculated to the bioreactors must be properly disposed of, generating additional costs. The nitrogen balance of the *P. stutzeri* UFV5 isolate showed that 47% of all ammonium removed is transformed into microbial biomass and 53% is lost as gaseous nitrogen, approximately. According to the literature, HN/AD microorganisms use approximately 40% of the ammonium removed for biomass, and 60% is transformed into gaseous nitrogen^10,17,18^. As HN/AD does not use ammonium as an electron source for energy production, we believe that this compound is toxic to them and its removal occurs as a mechanism of detoxification of the medium.

The intermediate compounds hydroxylamine, nitrite and nitrate were not detected in the medium, and due to the high sensitivity of the methodologies used, we believe that they were not detected because they were not present in the medium. The absence of these compounds can be explained possibly for two reasons: they may not have been produced, indicating that the route of HN/AD does not pass through these intermediates, or, for serving as a substrate for other enzymes, they may have been consumed before being detected.

The second part of this work aimed at investigating the enzymes that are involved in the HN/AD process through bioinformatics tools, such as comparative genomics, along with molecular biology, by PCR and analysis of the *P. stutzeri* UFV5 isolate transcriptome when the route of interest was induced. Comparative genomics were performed with sequences of enzymes known to be involved in the autotrophic nitrification and anaerobic denitrification processes against the genome of the *P. stutzeri* strain CGMCC 1.1803 deposited in the database, and PCR was performed with primers specific for the key enzymes of these processes, however, the genome used was from the *P. stutzeri* UFV5. Both methodologies showed that genes encoding the enzymes involved in autotrophic nitrification process are not present in the *P. stutzeri* genome, although those of anaerobic denitrification are present.

Daum and coworkers (1998)^19^ were able to purify and sequence the gene encoding the ammonia monooxygenase enzyme in the HN/AD *Pseudomonas putida* by hybridizing the DNA of this isolate with a probe specific for the gene that encodes the smaller subunit of this enzyme in *Nitrosomonas europaea*, an autotrophic nitrifying agent. They observed that the sequences have some similar and distinct regions, and that the HN/AD isolate ORF contained approximately 234 amino acids more than the conventional nitrifying ORF. This difference may reflect the non-detection of the amoA gene by methods based on known sequences.

The enzyme Hao - hydroxylamine oxidoreductase has been previously isolated and purified from HN/AD microorganisms: in 1993, Wehrfritz^20^ and coworkers purified this enzyme from the isolated *Thiosphaera pantotropha* and observed that it considerably differs from the same enzyme found in autotrophic nitrifying microorganisms. In addition, they observed that the hao enzyme they isolated was capable of using the periplasmic redox proteins, cytochrome c551 and pseudoazurin as electron receptors, which may also serve as electron donors for enzymes that perform the denitrification process, suggesting that hao is the enzyme responsible for coupling heterotrophic nitrification processes with aerobic denitrification. In 1996, the same group of researchers^21^, detected hao activity in species of *Pseudomonas* and *Aeromonas*, and found that the enzyme isolated from *Pseudomonas* species were very similar to the isolated form *Paracoccus denitrificans*, both capable of performing the HN/AD^22^. In this work we did not identify the gene encoding the hao enzyme in the *P. stutzeri* genome, either by comparative genomics or PCR, possibly because the hao enzyme from HN/AD differs considerably from the autotrophic nitrifying enzyme, and these analyzes are based on the known sequences of these enzymes, or simply because it is not present in the genome of this isolate.

Several species of the genus *Pseudomonas* are anaerobic denitrifier, which explains the presence of genes related to this process in *P. stutzeri*. The presence of the denitrification genes in the bacterial genome does not guarantee that the enzymes encoded by them participate in the HN/AD process. In addition, the absence of genes encoding the nitrification enzymes suggests that the HN/AD process is performed by another metabolic pathway with still unknown enzymes or by the same enzymes, but different enough not to be identified by the methodologies used in this study. To try to identify these enzymes, we chose to study the *P. stutzeri* UFV5 transcriptome in the HN/AD pathway induction condition.

To obtain and study the mRNA under HN/AD pathway induction conditions, these conditions were first standardized. It was observed that as the ammonium concentration increased, the consumption per unit of optical density ration also increased, showing that the effect was dose-dependent for the first 4 hours after incubation. Moreover, it was observed that the growth of *P. stutzeri* UFV5 was the same under both conditions, low and high ammonium concentration, with only ammonium consumption varying, which was higher when the ammonium concentration in the medium was 1.32 gL^−1^. This indicates that as the amount of ammonium increased, there was an increase in the removal pathway genes, which caused an increase in ammonium consumption. Another important factor is that this induction occurred in the initial 4 hours of the process, more precisely in the first 2 hours, where a higher amount of ammonium was consumed (Fig. 5). Having standardized the HN/AD pathway induction conditions, the *P. stutzeri* UFV5 isolate was inoculated into HNM medium at concentrations of 0.16 gL^−1^ and 1.32 gL^−1^ of ammonium, and two hours after inoculation, its mRNA was extracted for transcriptome study.

Principal component analyses of the transcriptome data showed that our results are reproducible, since the biological replicates were similar. The transcriptome showed that there was an increase in the expression of genes when the concentration of ammonium was increased, possibly being those involved in the HN/AD process, and that a greater number of genes had their expression positively regulated, indicating that an increase of the ammonium concentration was able to induce expression of specific genes that can be associated with the process.

No genes encoding the enzymes involved in conventional processes were differentially expressed under the HN/AD pathway induction conditions. As seen earlier, the autotrophic nitrification genes were not detected in the *P. stutzeri* genome, but those of anaerobic denitrification were, and if they were actually involved in the HN/AD process, their expression would have been differentiated. A study by Zhang^23^ and coworkers evaluated the proteomic profile of the isolate *Paracoccus versutus* KS293 during ammonium removal in aerobiosis and anaerobiosis, and the results show that in the aerobic condition no enzymes from conventional processes were expressed, corroborating the idea that other enzymes are part of this pathway of removal.

When searching for genes that are possibly involved in the pathways of the HN/AD process, among the 11 genes that were expressed differentially (Fig. 6A) with statistical significance in the condition of higher ammonium concentration, two have drawn attention: that which encodes the cellulase with the glycosyl hydrolase domain, and that which encodes the cytochrome c oxidase. When we align the amino acid sequences of the enzyme ammonia monooxygenase and the cellulase (glycosyl hydrolase family 5), we observe a region of alignment with highly conserved amino acids, suggesting that the catalytic domain of glycosyl hydrolase enzyme is also present in the ammonium monooxygenase enzyme, which performs the first step of autotrophic nitrification, the oxidation of ammonium to hydroxylamine (Fig. S3). The gene that encodes cytochrome c oxidase is directly involved in oxidation-reduction reactions, and the process of nitrification and denitrification takes place through successive oxidation and reduction reactions. Based on the results, these two genes are strong candidates for composing the HN/AD pathway of the *P. stutzeri* UFV5 isolate.

Among the 29 positively regulated genes (Fig. 6B), the second most expressed gene (second largest fold change) was the gene encoding the CsbD-like protein, which is a stress response protein, indicating that in addition to the CsbD-like protein being important in high ammonium concentration, this condition was stressful for *P. stutzeri* UFV5. Several genes have been noted to have unknown functions, including the third most expressed gene in the condition of high ammonium concentration (Fold change 8.3), and since we believe that the HN/AD process is performed by a metabolic pathway not yet elucidated, these genes may also be of interest for further investigation in the future. Among the genes differentially expressed exclusively in the condition of low ammonium concentration and those expressed in the two conditions that were negatively regulated, none had highlighted, and many of them were also of unknown function.

The functional correlation between genes that were positively regulated showed enrichment of genes encoding the Rps and Rpm family proteins, and according to the Pfam protein family database^24^, these proteins are ribosomal and are involved in the protein translation process, indicating that the higher ammonium concentration induced an increase in the protein expression of the translation. It was also possible to observe, with the enrichment analysis of cellular processes, that there was a change in the protein translation of the cell in the high ammonium concentration condition, indicating that excesses of extracellular ammonium were incorporated in amino acids for protein production. Moreover, an increase of the translation rate enables those key proteins associated with ammonium removal to be produced in high concentrations, even with no association with an increase of the transcriptional rate.

Although our experimental data were insufficient to define the HN/AD process pathway, the results of all our analyzes were corroborated by one another, showing that although genes encoding enzymes involved in autotrophic nitrification and anaerobic denitrification processes are used to study the HN/AD, they possibly do not participate in the pathway. Furthermore, *P. stutzeri* UFV5 isolated in this work is capable of HN/AD and removes ammonium with high efficiency in different carbon sources, C/N ratio, pH, salinity and temperature without excessive biomass production, presenting high potential for application in wastewater treatment plants. Given the importance of the HN/AD process for biological ammonium removal, the results obtained by the transcriptome are a starting point in the search for a molecular marker for these microorganisms as well as the elucidation of the complete pathway of removal.

## Conclusion

The bacterium *P. stutzeri* UFV5 characterized in this work was able to remove ammonium and produce nitrogen gas in aerobiosis, being able to perform the HN/AD process. Its response to the physicochemical factors influencing ammonium removal was promising: the different C/N ratios and temperatures evaluated did not affect the HN/AD process. Comparative genomics and amplification of the genes involved in the conventional processes of biological removal of ammonium showed that *P. stutzeri* UFV5 has no genes encoding the classic enzymes involved in nitrification, and since this bacterium is responsible for anaerobic denitrification, it has the genes for this process, suggesting that other enzymes are responsible for the HN/AD process. This hypothesis was corroborated by the transcriptome analysis of the *P. stutzeri* UFV5 isolate under conditions of HN/AD pathway induction, which showed that no gene related to conventional processes of ammonium removal was expressed. However, there was a change in the cell translation machinery when the ammonium concentration increased, indicating that *P. stutzeri* UFV5 expressed other genes, possibly related to stress caused by the high ammonium concentration. There was also a significant increase in the expression of different genes that encode proteins related to oxy-reduction reactions, which have high potential for being the enzymes involved in the HN/AD pathway, which may be targets for future studies to elucidate this pathway in *P. stutzeri* UFV5.

## Material and Methods

### Pseudomonas stutzeri UFV5

The bacterial strain used in this work, UFV5, was previously isolated from the activated sludge sample of an oil-effluent treatment plant by our research group and was deposited in the Brazilian Collection of Environmental and Industrial Microorganisms (CBMAI).

### Taxonomic identification of the isolate

One colony of the isolate was activated in 5 mL of liquid LB medium (gL^−1^ of distilled water: Tryptone 10.0, Yeast extract 5.0 and Sodium Chloride 10.0) (Difco™) and the culture was incubated at 30 °C under shaking at 150 rpm for 24 hours. The cells were pelleted by centrifugation and bacterial DNA was extracted as described by Pospiech & Neumann^25^. After extraction, the DNA was subjected to the polymerase chain reaction using primers 10 f and 1100r^26^ (sequences available in Table S5) for conserved regions of the 16S rRNA gene. The amplicon was purified and sequenced. The sequences obtained were compared to the 16S rRNA gene sequences of organisms represented in the Genbank database (http://www.ncbi.nem.nih.gov) using BLASTn. For the phylogenetic analyses, sequences of reference organisms (Table S6) were selected as well as sequences of heterotrophic nitrifying/aerobic denitrifying bacteria already described in the literature of the same genus as the bacterium identified in this work. The sequences were aligned using the ClustalX program^27^ and analyzed with MEGA 7.0.18 software^28^. The construction of the phylogenetic tree was done according to the neighbor-joining method^29^, with bootstrap values calculated from 1000 replicates. A sequence of an organism belonging to the Archaea domain was used as an outgroup. A phylogenetic tree using the maximum likelihood method was also constructed to show that the methodology does not interfere in the taxonomic classification of the isolate (Fig. S4).

### Evaluation of ammonium removal

The ammonium removal was evaluated every 24 hours over 7 days by colorimetric testing as described by Chaney and Marbach^30^ in standard HNM medium^31^, with some modifications (gL^−1^ of distilled water: (NH_4_)_2_SO_4_ 0.66, piruvato de sódio 5.28, KH_2_PO_4_ 0.5, Na_2_HPO_4_ 0.5, MgSO_4_.7H_2_O 0,20, NaCl 30.0 and 2 ml de solução de elemento traço, pH 7.5 Solução de element traço, gL^−1^ of distilled water: EDTA.2Na 57.1, ZnSO_4_.7H_2_O 3.9, CaCl_2_.2H_2_O 7.0, MnCl_2_.4H_2_O 1.0, FeSO_4_.7H_2_O 5.0, (NH_4_)_6_Mo_7_O_24_.4H_2_O 1.1, CuSO_4_.5H_2_O 1.6 and CoCl_2_.6H_2_O 1.6, pH 6.0).

The analyses were carried out in 96-well microplates, with each well containing 5 μl of the culture aliquot of the isolate in HMN medium, 100 μl of phenol reagent and 100 μl of hypochlorite reagent. The plates were incubated for 20 minutes at 39 °C, and then the absorbance was read at the wavelength of 630 nm in the Multiskan GO spectrophotometer (Thermo Scientific). As a positive control, 5 μl of nitrifying activated sludge cultured in HNM was added, and as a negative control, 5 μl of medium without inoculum was added. All analyses were performed in triplicate.

### Validation of the HN/AD process

To validate the occurrence of heterotrophic nitrification/aerobic denitrification in *P. stutzeri* UFV5, the production of NO, N_2_O and N_2_ gases was monitored by gas chromatography (GC). One and a half milliliters of the isolate was inoculated into 15 ml of standard HNM medium, contained in 30 ml flasks. The flasks were hermetically sealed and the atmosphere inside them was saturated with oxygen (99.5% purity). The flasks were incubated at 30 °C with shaking at 150 rpm. The negative control was done using HNM medium without inoculum, and the positive control used the nitrifying activated sludge inoculated in HNM medium. As a positive control of the technique and guarantee of column operation, atmospheric air was injected into the chromatograph and the nitrogen gas peak was evaluated. Gas samples were collected at the end of 10 days using a 1 ml gas collection syringe. A 100 µl volume of gas was applied to a mass spectrometer coupled to a gas chromatograph (Agilent Gas Chromatograph, model HP-6890, coupled to an Agilent mass selector detector, model HP-5975) equipped with an HP-Plot molecular sieve 5 A column (30 m × 0.32 mm × 25 μm) (Sigma Aldrich). The procedure was performed according to Yao *et al*.^32^. Analyses were performed in triplicate.

### Physicochemical factors in ammonium removal

The effects of physicochemical factors were evaluated individually, so the medium composition and cultures incubation conditions were set so that only the carbon source or the C/N ratio or ph or temperature or salinity were variable.

A colony of the isolate was first activated in 5 ml of LB medium, and then, 100 μL of the inoculum was transferred to plastic tubes of 50 ml containing 10 ml of HNM medium. The standard incubation condition of the cultures was 30 °C under agitation at 150 rpm for 72 hours, and every 24 h the optical density of the isolate was read at the 600 nm wavelength, pH was measured and the ammonium removal analysis was done by the colorimetric method previously described.

The first factor evaluated was the carbon sources (sodium pyruvate, sucrose, sodium acetate, sodium citrate, sodium succinate and glucose), and the best source was used for all other conditions evaluated and for all subsequent analyzes after the physicochemical characterization. The other conditions evaluated were: C/N ratio = 4, 6, 8, 10 and 12; pH = 3, 5, 7 and 9; temperature = 20, 25, 30, 35 and 40 °C; and salinity = 0, 3, 6, 9, 12 and 15% NaCl. For all conditions evaluated, HNM medium without inoculum was used as negative control. All analyses were done in triplicate.

### Nitrogen balance

Nitrogen balance was done in 1 liter Erlenmeyers containing 250 ml of HNM medium with sodium citrate as carbon source. The colonies of *P. stutzeri* UFV5 were activated in 150 ml of LB medium, and a 10% (v/v) inoculum was transferred to 250 ml of HNM medium and incubated at 30 °C under agitation of 150 rpm for 72 hours. Every 24 hours, measurements of optical density, ammonium, hydroxylamine, nitrite and nitrate were made. At the end of 72 hours, the biomass nitrogen was calculated. All experiments were done in triplicate.

Cell growth was monitored by measuring the optical density at a wavelength of 600 nm in a Multiskan GO spectrophotometer (Thermo Scientific). The ammonium analysis was done by colorimetry, as described above. Hydroxylamine was determined according to Frear and Burrell^33^. Nitrite was quantified by the photometric method of N-(1-naphthalene)-diaminoethane, and nitrate by the hydrochloric acid method, described according to the Standard Methods manual of the APHA^34^. To calculate the amount of biomass nitrogen at the end of 72 hours, the 250 ml of culture was centrifuged at 6.000 g for 10 minutes, and the precipitate was oven-dried at 55 °C to a constant weight. Then, intracellular nitrogen was measured by the Kjeldahl method^35^. The amount of nitrogen gas was determined by the difference in relation to the values found for hydroxylamine, nitrite, nitrate and intracellular nitrogen, considering 100% of ammonium removal.

### Comparative genomics

As *P. stutzeri* UFV5 identified in this work has not yet had its genome sequenced, a comparative analysis was performed with the complete genome of *P. stutzeri* strain CGMCC 1.180330^9^ available at the NCBI (National Center for Biotechnology Information), as well as the nucleotide sequences of all the enzymes known to be involved in the ammonium removal process available in KEGG (Kyoto Encyclopedia of Genes and Genomes)^36^. All downloaded sequences were aligned by ClustalW^27^. Obtaining a criterion of 20% identity and 25% coverage, possible candidates for proteins in the genome of *P. stutzeri* that may be exerting the same function as enzymes known to be involved in the ammonium removal were selected, and the presence of protein domains associated with the ammonium removal process was validated using the Pfam database version 32.0^37^.

### Polymerase chain reaction

The genomic DNA of *P. stutzeri* UFV5 was subjected to amplification for the detection of genes encoding the enzymes related to the conventional nitrification and denitrification processes. The genes analyzed were: amo = ammonium monooxygenase^38^; hao = hydroxylamine oxide reductase^39^; nap = periplasmatic nitrate reductase^40^; nirS = nitrite reductase (cytochrome cd1 nitrite reductase) and nirK = nitrite reductase (copper nitrite reductase)^41^; norB = nitric oxide reductase^41^; and nosZ = nitric oxide reductase^41^. The sequences of the primers are available in Table S5. Amplicons were observed on 1% agarose gel (Fig. S5).

### Standardization of the conditions for the transcriptome study

To assess the significant difference in the expression of the genes involved in HN/AD, it was first checked whether the removal effect was affected by different concentrations of ammonium, indicating an induction mechanism. For this, the isolate was inoculated in 50 ml plastic tubes (Falcon) containing 10 ml of HNM medium with sodium citrate as carbon source, with different concentrations of ammonium (0.16, 0.33, 1.32 and 3.3 gL^−1^), with an initial optical density of the inoculum of 0.1, and the cultures were incubated at 30 °C under agitation at 150 rpm for 10 hours. Every 2 hours, optical density and ammonium removal were measured. After obtaining the results, the amount of ammonium consumed every two hours was divided by the optical density of the culture in the same time interval to normalize the data.

### HN/AD pathway induction

Using standardized conditions, two colonies of the isolate *P. stutzeri* UFV5 were activated in 15 ml of LB medium. An aliquot was transferred to Erlenmeyers containing 50 ml of HNM medium with sodium citrate as carbon source, with 0.16 gL^−1^ and 1.32 gL^−1^ of ammonium, in triplicate, with an initial optical density of 0.1. Cultures were incubated at 30 °C under agitation at 150 rpm for 2 hours (time determined in standardization). After this time, ammonium removal and optical density were measured. Aliquots of 1 ml of culture were taken for counting viable and non-viable cells with the Propidium Iodide dye on the flow cytometer (BD FACS Verse, Biosciences). Cells were pelleted at 6.000 g for 10 minutes, and RNA extraction was done with the QIAzol®lysis reagent (Qiagem) according to the manufacturer’s instructions. The extracted RNA was quantified, and treated with RNAse-free DNAse (Promega). The treated RNA was visualized on 1% agarose gel, lyophilized, and sent for RNAseq sequencing at Molecular Research DNA (www.mrdnalab.com, Shallowater, TX, USA) by the Illumina HiSeq platform.

### RNAseq data analyses

The pipeline used to analyze the data generated by RNAseq was performed according to the work of Conesa and coworkers^42^. Initially, the sequences were submitted to a quality analysis using the FastQC tool. The low-quality sequences containing nucleotides with Phred quality below 15 and the adapters were removed using the Trimmomatic tool^43^. The reference genome of the species *Pseudomonas stutzeri* deposited in the NCBI, NC_015740.1, was indexed through the Bowtie-build algorithm implemented in the Bowtie2 tool^44^, and the high-quality sequences from the transcriptome were aligned against it using the Tophat2 tool^45^. The differential accumulation of transcripts between the studied conditions was evaluated by the likelihood ratio test through the Cuffdiff algorithm implemented in the Cufflinks package^46^, with a p-value threshold of 0.001. The differentially expressed transcripts in the concentrations of ammonium at 0.16 gL^−1^ and 0.32 gL^−1^ were submitted to a search in the Uniprot and Pfam database for functional annotation. A protein-protein interaction network based on differentially expressed genes was done through the STRING database version 11^47^ with 0.7 of confidence parameter. Biological processes that were enriched in the network were identified by the BINGO plugin^48^ implemented in Cytoscape 3.7.1^49^, using the whole predicted protein based on the genome as a reference set. Before enrichment analysis, the GO terms associated with each protein were retrieved using the GOanna software^50^ for a search against the Uniprot database release 2018_11^51^, expected value of 10^−5^, word size of 3, low-complexity filter turned on, and using the BLOSUM62 matrix.

### Statistical analyzes

Applying the Shapiro-Wilk statistical normality test, it was found that all data sets followed normal distribution, and therefore all groups were compared by analysis of variance (ANOVA), followed by a post-hoc Tukey test. All statistical analyses were performed in MiniTab® 17.1.0 (Minitab, Inc., Quality Plaza, 1829 Pine Hall Road, State College, Pennsylvania, 16801, USA). Values with P ≤ 0.05 were considered to indicate statistical difference at 95% of confidence. All analyses were performed in triplicate.

For the transcriptome data, all statistical analyses were performed by R language 3.6.0^52^ implemented in the R-Studio software, where a t-test for comparison of two samples was applied, based on differentially expressed genes. The replicates were evaluated by main component analysis (PCA) implemented the R in software. Values with P ≤ 0.001 were considered to indicate statistical difference to 99.9% confidence.

## Supplementary information

Suppementary Information.

## Data Availability

The datasets generated during and/or analyzed during the current study are available in the NCBI Sequence Read Archive (SRA) repository, under accession number SRP150008.
