# Supplementary material for: Physicochemical characterization of Pseudomonas stutzeri UFV5 and analysis of its transcriptome under heterotrophic nitrification/aerobic denitrification pathway induction condition
Source: Sci Rep. 2020 Feb 10;10:2215. doi: 10.1038/s41598-020-59279-7 (PMC7010759; doi:10.1038/s41598-020-59279-7)
Supplement: Supplementary file 1 — Suppementary Information. [file 41598_2020_59279_MOESM1_ESM.pdf]

SUPPLEMENTARY INFORMATION

Physicochemical characterization of *Pseudomonas stutzeri* UFV5 and analysis of its transcriptome under heterotrophic nitrification/aerobic denitrification pathway induction condition.

Lívia Carneiro Fidélis Silva[1], Helena Santiago Lima[1], Tiago Antônio de Oliveira Mendes[2], Adilson Sartoratto[3], Maira Sousa[4], Rodrigo Suhett de Souza[4], Sérgio Oliveira de Paula[5], Valéria Maia de Oliveira[3], Cynthia Silva[1,*]

[1]Department of Microbiology, Federal University of Viçosa, Viçosa, Minas Gerais, 36570-900, Brazil.

[2]Departament of Biochemistry, Federal University of Viçosa, Viçosa, Minas Gerais, 36570-900, Brazil.

[3]Pluridisciplinary Center for Chemical, Biological and Agricultural Research, Campinas State University, Campinas, São Paulo, 13083-970, Brazil.

[4]Petrobras - Research and Development Center (CENPES), Petrobras, Rio de Janeiro, 21941-915, Brazil.

[5]Department of General Biology, Federal University of Viçosa, Viçosa, Minas Gerais, 36570-900, Brazil.

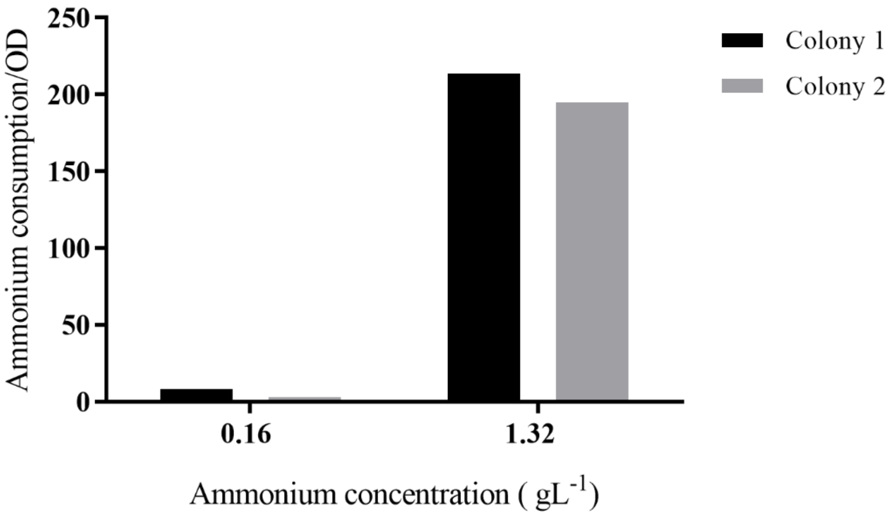

Figure S1. Ammonium consumption by optical density unit of the two colonies of the isolate *P. stutzeri* UFV5 inoculated in HNM medium with low and high ammonia concentration after 2 hours incubation at 30 °C.

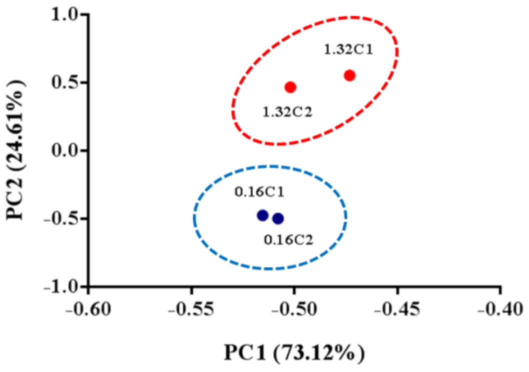

Figure S2. Principal Components Analysis of transcriptome data obtained from biological replicates of *P. stutzeri* UFV5 isolate inoculated in HNM medium with low and high ammonium concentration.

```
CLUSTAL O(1.2.4) multiple sequence alignment

WP_013982284.1      ------MSTNLF------TG----------------------------------------      8
2_K10535_neu        MRIGEWMRGLLLCAGLMMCGVVHADISTVPDETYDALKLDRGKATPKETYEALVKRYKDP      60
                          *   *:       *

WP_013982284.1      ---ARK----ALVASIAMAALMGGVTVA--------------------------------      29
2_K10535_neu        AHGAGKGTMGDYWEPIAISIYMDPNTFYKPPVSPKEVAERKDCVECHSDETPVWVRAWKR      120
                       * *         **::  *.  *.

WP_013982284.1      ------------------------------------------------------------      29
2_K10535_neu        STHANLDKIRNLKSDDPLYYKKGKLEEVENNLRSMGKLGEKETLKEVGCIDCHVDVNKKD      180

WP_013982284.1      --------TVPYAAAATTVAAASVSTKVNAFTNTDWLNGVWRTGAGFSIPATAANQAAFK      81
2_K10535_neu        KADHTKDIRMPTADTCGTCHLREFAERESERDTMVWPNGQWPAGRPSHALDYTAN-----      235
                             :* * :. *    ..: : .   .  * ** * :*        :**

WP_013982284.1      AGASVRLADGQVRTISRAQVVGSNMSVFLDGAKLDGNKVGAPQSVATVAAAPAPSPAPSA      141
2_K10535_neu        ------------------------------------------------------------      235

WP_013982284.1      PSTTNGKPLLVGVNLSGAGFGPSVVPGTHGTNYTYPAESYYKKYADLGMPLVRLPFLWER      201
2_K10535_neu        ------------------------------------------------------------      235

WP_013982284.1      IQPKLNTPLNATELARLKQSLDFAQKHNVKVILDLHNYYRYFNKLIGSNEVPISSFAAVW      261
2_K10535_neu        ------------------------------------------------------IETTVW      241
                                                                        ::**

WP_013982284.1      KQIAQEVVNHPAVEGYGLMNE-----------------------------PHSTNGLWPQ      292
2_K10535_neu        AAMPQREVA----EGCTMCHTNQNKCDNCHTRHEFSAAESRKPEACATCHSGVDHNNWEA      297
                      : *. *     **  : :                                  :. *

WP_013982284.1      AALAAAQAIRTVDSKRWIYVA--------------------------------GDRWSS      319
2_K10535_neu        YTMSKHGKLAEMNRDKWNWEVRLKDAFSKGGQNAPTCAACHMEYEGEYTHNITRKTRWAN      357
                     :::    :  :: .:* : .                                   **:.

WP_013982284.1      AFHWPHYNTQLISNPWMRDPKNNLVYEAHMYIDKDFSGNYFDKNEKFDPMIGVNRV---K      376
2_K10535_neu        YPFVPGI-AENITSDWSEARLDSWVLTCTQCHSERFARSYLDLMDKG-TLEGLAKYQEAN      415
                      . *   :: *:. * .   :. *  .    .: *: .*:*  :*   : *: :    :

WP_013982284.1      PFVD-WLKQNKLRGYIG------EHGIPDFS------------P-----SALVATDNLLS      412
2_K10535_neu        AIVHKMYEDGTLTGQKTNRPNPPEPEKPGFGIFTQLFWSKGNNPASLELKVLEMAENNLA      475
                     :*.   ::..* *         *   *.*.            *     ..*  ::* *:

WP_013982284.1      Y--LRQNCIPSTYWAAGPWWGEYALSLDVTSGKH--RPQLPILQKHAK------------      456
2_K10535_neu        KMHVGLAHVNPGGWTYTEGWGPMNRAYVEIQDEYTKMQELSALQARVNKLEGKQTSLLDL      535
                       :    :    *:    **    :    ..::    :*  ** :.:

WP_013982284.1      -TAHSCTSIGPL----------------------- 467
2_K10535_neu        KGTGEKISLGGLGGGMLLAGALALIGWRKRKQTRA 570
                      : .  *:* *
```

Figure S3. Alignment of the amino acid sequence of the ammonia monooxygenase - amoA enzyme of autotrophic nitrifying microorganism with the translated sequence of the gene encoding a glycosyl hydrolase family 5, positively regulated during the HN/AD pathway. * positions - fully conserved residue; : positions – conservation between amino acid groups of similar properties and . positions - conservation between amino acid groups of weakly similar properties.

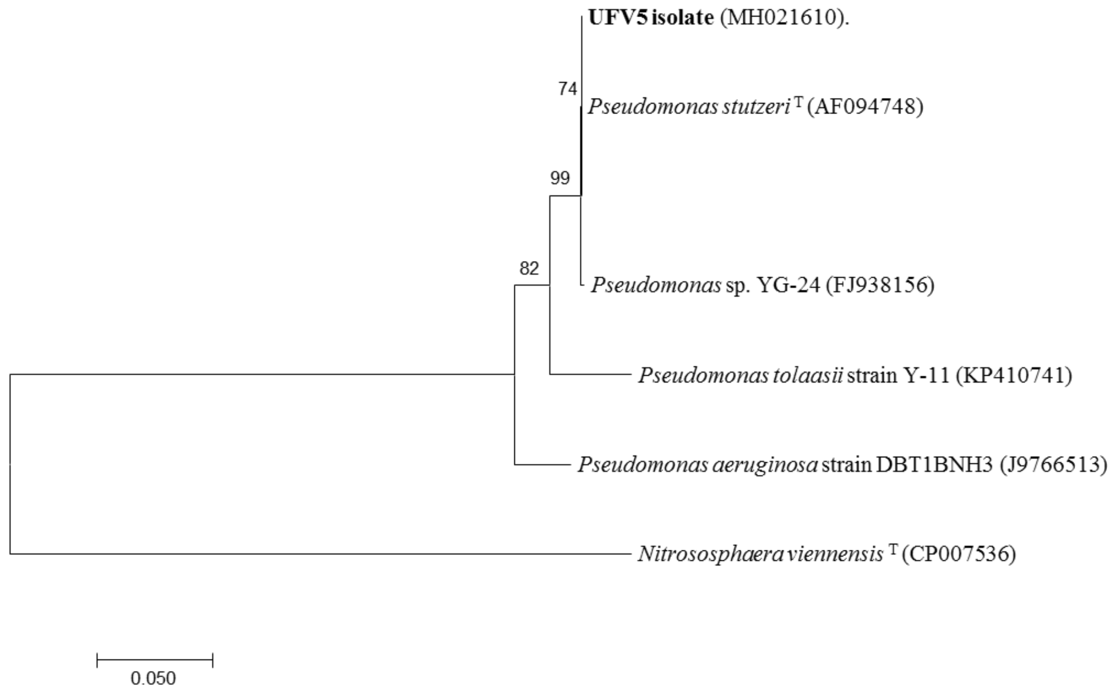

Figure S4. Phylogenetic tree based on the partial sequence of the 16S rRNA of the UFV5 isolate, reference sequence (T) and sequences of HN/AD microorganisms belonging to the same genus described in the literature. The tree was constructed by the maximum likelihood method with a bootstrap value represented in the branches referring to 1000 replications. The accession numbers of the GenBank sequences are shown in parentheses. The *Nitrososphaera viennensis* sequence was added as an outgroup.

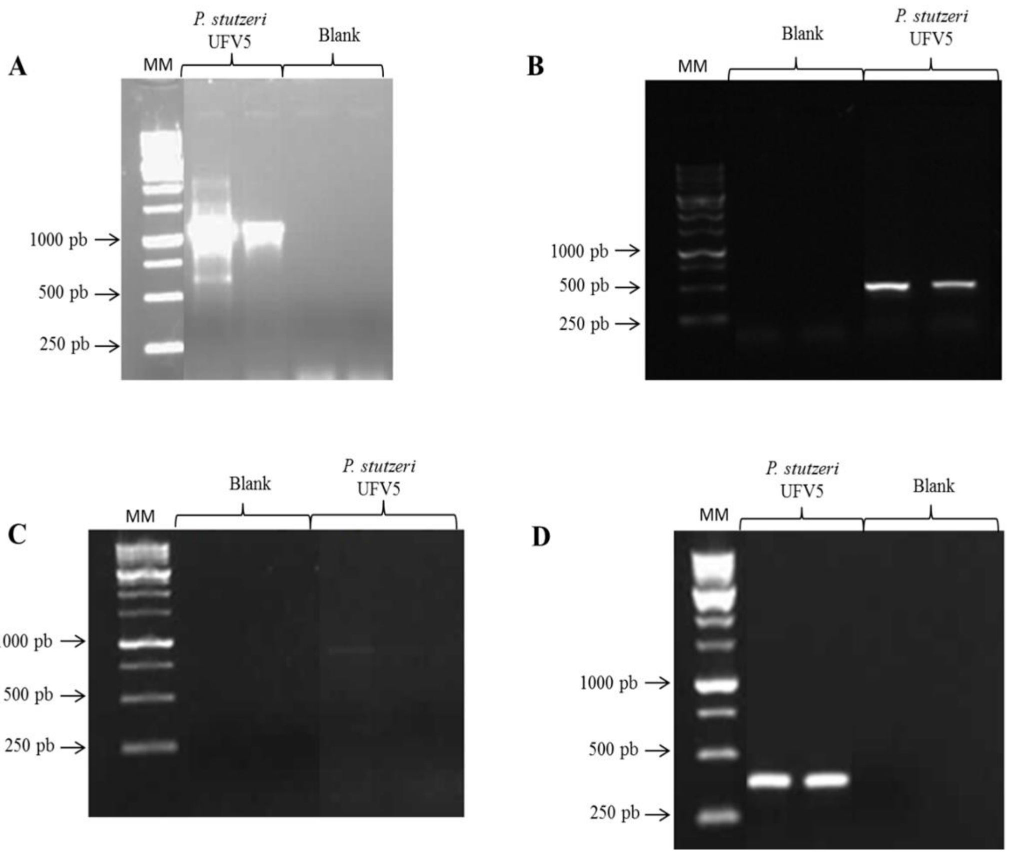

Figure S5. Amplification results of genes encoding enzymes related to the conventional ammonium removal process in *P. stutzeri* UFV5 A) gene encoding the enzyme periplasmatic nitrate reductase - Nap (876 bp); B) gene encoding the enzyme nitrite reductase - NirS (425 bp); C) gene encoding the enzyme nitric oxide reductase - NorB (669 bp); and D) gene encoding the enzyme nitrous oxide reductase - NosZ (300 bp). MM = 1 Kb molecular marker.

Table S1. Growth response profile of the isolate *P. stutzeri* UFV5 when submitted to different carbon sources, C/N ratio, pH, salinity and temperature after 72 hours of incubation in HNM medium. The values shown represent the mean of the optical density in each condition, followed by the standard deviation (SD). To each condition, means followed by at least one same letter did not differ at the 5% level of significance by the Tukey test.

Physicochemical factors		***P. stutzeri* UFV5**	
		**Mean**	**SD**
**Carbon Source**	Sodium Piruvate	1,45a	0,01
	Sucrose	1,03e	0,04
	Sodium Acetate	1,19c	0,01
	Sodium Citrate	1,34b	0,01
	Sodium Succinate	1,09de	0,01
	Glucose	1,16cd	0,06
**C/N Ratio**	4	1,01e	0,01
	6	1,23d	0,01
	8	1,35b	0,02
	10	1,54a	0,01
	12	1,29c	0,01
**pH**	3	0,10c	0,00
	5	0,10c	0,00
	7	1,35a	0,02
	9	1,31b	0,01
**Salinity (%)**	0	1,12c	0,01
	3	1,35b	0,02
	6	1,49a	0,02
	9	0,10d	0,00
	12	0,10d	0,00
	15	0,03e	0,00
**Temperature (°C)**	20	1,42a	0,01
	25	1,43a	0,02
	30	1,35b	0,02
	35	1,29c	0,01
	40	1,32b	0,01

Table S2. Genes that were expressed exclusively in the condition of lower concentration of ammonium ($0.16\ gL^{-1}$) in the isolate *P. stutzeri* UFV5 during HN/DA process. The reference proteome used for functional annotation of genes was from *P. stutzeri* strain CGMCC 1.180330

Gene code in reference proteome	Annotation
WP_013982075.1	L-aspartate oxidase
WP_013982191.1	Cobalamin biosynthesis protein
WP_013982295.1	Glycosyltransferase family 1 protein
WP_013982302.1	Recombinase reca
WP_011912701.1	Elongation factor Ts
WP_013982344.1	2-C-methyl-D-erythritol 4-phosphate cytidylyltransferase
WP_013982569.1	Cbb3-type cytochrome oxidase assembly protein ccos
WP_003294837.1	Ccoq/fixq family Cbb3-type cytochrome c oxidase assembly chaperone
WP_013982665.1	Methyl-accepting chemotaxis protein
WP_013982668.1	FAD-dependent oxidoreductase
WP_041771932.1	Universal stress protein uspa
WP_011913361.1	DUF2970 domain-containing protein
WP_011913499.1	Deoxyguanosinetriphosphate triphosphohydrolase
WP_011913506.1	DUF2835 domain-containing protein
WP_013983036.1	DUF2797 domain-containing protein
WP_013983299.1	Hypothetical protein
WP_079327710.1	Hypothetical protein
-	Trna-Thr
WP_013983492.1	Biotin attachment protein
WP_013983516.1	KTSC domain-containing protein
WP_011914066.1	Hypothetical protein
WP_011914160.1	Hypothetical protein
WP_011914232.1	Hypothetical protein
WP_013983773.1	50S ribosomal protein L25
-	Trna-Leu
WP_013983882.1	Carbamoyl-phosphate synthase small subunit
WP_013983964.1	FTR1 family iron permease

WP_013983965.1	Anaerobic ribonucleoside-triphosphate reductase activating protein
WP_013983996.1	ABC transporter permease
WP_080565002.1	Hypothetical protein
WP_013984149.1	DUF2789 domain-containing protein
WP_003286662.1	Yqae/Pmp3 family membrane protein
WP_013984285.1	Hypothetical protein
WP_013984335.1	Sulfur carrier protein this
WP_011915081.1	Glutaredoxin 3
WP_011915106.1	Amino acid ABC transporter
WP_013984507.1	Twin transmembrane helix small protein
WP_013981211.1	M48 family peptidase
WP_049790892.1	MFS transporter
-	Trna-Thr
WP_011912000.1	50S ribosomal protein L17
WP_013981731.1	DUF2796 domain-containing protein
WP_013981233.1	Polyamine ABC transporter substrate-binding protein
WP_013981783.1	Alcohol dehydrogenase adhp
WP_013981788.1	HAD family hydrolase
WP_020306566.1	DUF2256 domain-containing protein
-	Trna-Ser
WP_013982800.1	DUF2790 domain-containing protein
WP_013981345.1	Hypothetical protein
WP_003290130.1	General stress protein
WP_003290130.1	Hypothetical protein
WP_003303200.1	Ferredoxin

Table S3. Genes that were expressed exclusively in the condition of higher concentration of ammonium (1.32 gL^{-1}) in the isolate *P. stutzeri* UFV5 during HN/DA process. The reference proteome used for functional annotation of genes was from *P. stutzeri* strain CGMCC 1.180330

Gene code in reference proteome	Annotation
WP_011912316.1	Type III effector
WP_011912368.1	DUF1289 domain-containing protein
WP_013982078.1	Positive regulator for alginate biosynthesis mucc
WP_013982080.1	Elongation factor 4
WP_014596052.1	Hypothetical protein
WP_007146183.1	Transcriptional regulator
WP_013982263.1	Hypothetical protein
WP_013982338.1	Acetyl-coa carboxylase carboxyltransferase subunit alpha
WP_013982357.1	RNA polymerase sigma factor rpos
WP_011913021.1	Dihydrolipoyl dehydrogenase
WP_013982738.1	Hypothetical protein
WP_013982968.1	DNA gyrase subunit A
WP_003285516.1	Hypothetical protein
WP_013983116.1	Integration host factor subunit beta
WP_013983775.1	Redox-regulated atpase ychf
WP_013984074.1	Hypothetical protein
WP_013984287.1	Hemerythrin domain-containing protein
WP_013984443.1	Hypothetical protein
WP_013984546.1	F0F1 ATP synthase subunit delta
WP_011911694.1	Rida family protein
WP_011911719.1	Cytochrome c5 family protein
WP_013981656.1	3-dehydroquinate synthase
WP_011911988.1	50S ribosomal protein L4
WP_013981769.1	MFS transporter
WP_013981841.1	Hypothetical protein
WP_013981925.1	LPS export ABC transporter ATP-binding protein
WP_013981929.1	Phospholipid ABC transporter ATP-binding protein mlaf

WP_003287302.1	50S ribosomal protein L13
WP_011912294.1	DUF2007 domain-containing protein
WP_013982399.1	Hypothetical protein
WP_041771694.1	Trna dihydrouridine(20/20a) synthase dusa
WP_011912882.1	Acetyl-coa C-acyltransferase fada
WP_011912949.1	50S ribosomal protein L31 type B
WP_011913018.1	Succinate dehydrogenase hydrophobic membrane anchor protein
WP_013982642.1	Hypothetical protein
WP_011913168.1	Bifunctional phosphoserine phosphatase/homoserine phosphotransferase thrh
WP_013981380.1	Diaminobutyrate--2-oxoglutarate transaminase
WP_013983117.1	RNA helicase
WP_013983240.1	Electron transfer flavoprotein subunit beta/fixa family protein
WP_013981402.1	Glsb/yeaq/ymge family stress response membrane protein
WP_013983585.1	Hypothetical protein
WP_013983603.1	Hypothetical protein
WP_013983649.1	Iscs subfamily cysteine desulfurase
WP_013983712.1	DUF2188 domain-containing protein
WP_013983714.1	Hypothetical protein
WP_013983723.1	Amino acid transporter
WP_013983754.1	Hypothetical protein
-	Trna-Met
WP_013981481.1	Outer membrane protein assembly factor bame
WP_013983997.1	ABC transporter ATP-binding protein
WP_013984167.1	Ribosome alternative rescue factor arfa
WP_017245397.1	DUF4212 domain-containing protein
WP_003282841.1	DUF1328 domain-containing protein
WP_013984545.1	F0F1 ATP synthase subunit alpha
WP_041771624.1	Hypothetical protein
WP_003295433.1	DNA-directed RNA polymerase subunit omega
WP_013981620.1	Hypothetical protein
WP_003286066.1	Hypothetical protein
WP_011911989.1	50S ribosomal protein L2

WP_011911990.1	30S ribosomal protein S17
WP_013981732.1	Hypothetical protein
WP_041771637.1	Pepsy domain-containing protein
WP_017245997.1	Hypothetical protein
WP_013981931.1	Toluene tolerance protein
WP_003283978.1	Global regulator protein family
WP_013982284.1	Cellulase (glycosyl hydrolase family 5)
WP_013982284.1	Pilz domain-containing protein
WP_013982773.1	Phospholipase_D-nuclease N-terminal
WP_003298841.1	Outer membrane lipoprotein carrier protein lola
WP_013982942.1	Cold shock domain protein cspd
WP_003293422.1	Cold-shock protein
WP_013983347.1	Tetr family transcriptional regulator
WP_013983644.1	Iron-sulphur cluster assembly
WP_013983984.1	Prokaryotic Cytochrome C oxidase subunit IV
WP_011914916.1	Hypothetical protein
WP_003290642.1	30S ribosomal protein S21
WP_011912146.1	50S ribosomal protein L27

Table S4. Differentially expressed genes shared by the two evaluated ammonium conditions that were positively or negatively regulated during the HN/AD process. The reference proteome used for functional annotation of genes was from *P. stutzeri* strain CGMCC 1.180330.

Gene code in reference proteome	Annotation
WP_013982487.1	Zinc chelation protein
WP_011913743.1	Hypothetical protein
-	Trna-Val
-	Trna-Ser
WP_013983425.1	Transcriptional regulator
WP_013981442.1	Cellulose biosynthesis protein bcsf
WP_013983650.1	Fe-S cluster assembly transcriptional regulator iscr
WP_014597954.1	Hypothetical protein
WP_011915051.1	C4-dicarboxylate ABC transporter substrate-binding protein
WP_020306711.1	DUF4880 domain-containing protein
WP_011911979.1	50S ribosomal protein L10
WP_003281581.1	Hypothetical protein
WP_013981933.1	Bola family transcriptional regulator
WP_013982219.1	Hypothetical protein
WP_013983293.1	DUF1127 domain-containing protein
WP_013983442.1	Outer membrane porin oprd family
WP_013983446.1	Hypothetical protein
WP_013983969.1	U32 family peptidase
WP_003283098.1	RNA-binding protein Hfq
WP_011915136.1	Hypothetical protein
-	6s rna
WP_011912321.1	Flavodoxin
WP_013982049.1	Cytochrome c assembly protein
WP_079329368.1	DNA or RNA helicase of superfamily II
WP_013982219.1	Hypothetical protein
WP_013982276.1	Hypothetical protein
WP_013982940.1	Translation initiation factor IF-1

WP_079328957.1	Hypothetical protein
WP_003290924.1	50S ribosomal protein L34
WP_003301052.1	50S ribosomal protein L31
WP_003281818.1	50S ribosomal protein L30
WP_041771656.1	DNA gyrase inhibitor yacg
WP_011911573.1	Twin-arginine translocase tata/tate family subunit
WP_013982009.1	DNA-binding protein
WP_013982024.1	Hypothetical protein
WP_011912373.1	30S ribosomal protein S16
WP_011912416.1	DUF2905 domain-containing protein
WP_003282211.1	Cold-shock protein
WP_013982283.1	DUF2945 domain-containing protein
WP_013982298.1	Csbd family protein
WP_013982320.1	Hypothetical protein
WP_041771612.1	50S ribosomal protein L28
WP_003284185.1	50S ribosomal protein L33
WP_013982479.1	DUF1653 domain-containing protein
WP_011912999.1	Sulfurtransferase tusa
WP_003294452.1	Alanine-zipper, outer membrane lipoprotein
WP_011913223.1	Hypothetical protein
WP_003298367.1	50S ribosomal protein L35
WP_011913490.1	Peptidase inhibitor I78 family protein
WP_013983005.1	Unfunctional protein
WP_011913504.1	Ribosome modulation factor
WP_002552845.1	Methylthioribulose-1-phosphate dehydratase
-	Signal recognition particle srna small type
WP_011913664.1	Heme exporter ccmd
WP_003283670.1	NADH dehydrogenase
WP_003293518.1	50S ribosomal protein L32
WP_041771783.1	(Na+)-NQR maturation nqrm
WP_011913772.1	Hypothetical protein
WP_013983373.1	(2Fe-2S)-binding protein

WP_026006463.1	Putative quorum-sensing-regulated virulence factor
WP_003300856.1	DUF3079 domain-containing protein
WP_003246255.1	Cold-shock protein
WP_013984129.1	DUF2788 domain-containing protein
WP_011911750.1	Phosphate-starvation-inducible E
WP_003281834.1	50S ribosomal protein L29
WP_003281814.1	50S ribosomal protein L36
WP_011912887.1	Hypothetical protein
WP_013983044.1	Hypothetical protein
WP_011911422.1	DUF465 domain-containing protein
WP_013983537.1	Hypothetical protein
WP_011914531.1	Oxidoreductase
WP_003283112.1	30S ribosomal protein S18
WP_003284846.1	F0F1 ATP synthase subunit C

Table S5. List of primers used in this study.

Primer	Sequence (5'-3')	Target gene	Reference
10-f	GAGTTTGATCCTGGCTCAG	16S rDNA	Lane et al., 1985
1100-r	AGGGTTGCGCTCGTTG		
amoA-1f	GGGGTTTCTACTGGTGGT	ammonia monooxygenase	Rotthauwe et al., 1997
amoA-2r	CCCCTCKGSAAAGCCTTCTTC		
hao-f1	TGCGTGGARTGYCAC	hydroxylamine oxide reductase	Yang et al., 2015
hao-f3	AGRTARGAKYSGGCAAA		
nap1	TCTGGACCATGGGCTTCAACCA	periplasmatic nitrate reductase	Zhu et al., 2012
nap2	ACGACGACCGGCCAGCGCAG		
nirS-f	GTSAACGTSAAGGARACSGG	nitrite reductase cd1	Wan et al., 2011
nirS-r	GASTTCGGRTGSGTCTTGA		
nirK-f	GGMATGGTKCCSTGGCA	nitrite reductase	Wan et al., 2011
nirK-r	GCCTCGATCAGRTTRTGG		
norB-f	CGNGARTTYCTSGARCARCC	nitric oxide reductase	Wan et al., 2011
norB-r	CRTADGCVCCRWAGAAVGC		
nosZ-f	CCCGCTGCACACCRCCTTCGA	nitrous oxide reductase	Wan et al., 2011
nosZ-r	CGTCGCCSGAGATGTCGATCA		

*R: A/G; Y: C/T; M: A/C; K: G/T; S: C/G

Table S6. Accession number of the sequences used in this work deposited in GenBank.

**Microorganisms**	**GenBank acession number**
Pseudomonas stutzeri UFV5	MH021610
Pseudomonas stutzeri tipe strain	AF094748
P. stutzeri strain CGMCC 1.1803	NC_015740.1
Pseudomonas stutzeri strain P3	FJ869912
Pseudomonas sp. YG-24	FJ938156
Pseudomonas tolaasii Y-11	KP410741
Pseudomonas aeruginosa strain DBT1BNH3	J9766513
Nitrososphaera viennensis tipe strain	CP007536
